# Red Cell Distribution Width, Mean Platelet Volume, and Neutrophil/Lymphocyte Ratio in Patients With Irritable Bowel Syndrome

**DOI:** 10.7759/cureus.44496

**Published:** 2023-08-31

**Authors:** Bahadır Kartal, Ibrahim Tayfun Sahiner

**Affiliations:** 1 General Surgery, Hitit University, Çorum, TUR; 2 General Surgery, Hitit University, Faculty of Medicine, Çorum, TUR

**Keywords:** lymphocyte count, neutrophil count, rdw, mpv, inflammation, irritable bowel syndrome

## Abstract

Introduction

Irritable bowel syndrome (IBS) is a common functional gastrointestinal disorder characterized by persistent abdominal pain and variable bowel patterns, impacting individuals' quality of life. Despite its functional nature, recent research has indicated the role of inflammatory processes in IBS development. This study aims to investigate the potential diagnostic value of routine blood parameters and their relationship with IBS.

Methods

In this retrospective analysis, patients diagnosed with IBS based on the ROME IV criteria were identified from the outpatient clinic of Hitit University Erol Olçok Teaching and Research Hospital between January 1, 2023, and May 1, 2023. Exclusion criteria encompassed specific medical conditions, psychiatric disorders, and organic bowel pathologies. A cohort of 100 IBS patients and 100 healthy controls were included for comparison. Comprehensive blood data, including neutrophil count, lymphocyte count, hemoglobin level, red cell distribution width (RDW), mean corpuscular volume (MCV), mean platelet volume (MPV), and platelet count, were collected. Statistical analyses were conducted using SPSS for Windows version 26.0 (IBM Corp., Armonk, NY). Descriptive statistics, Pearson's or Spearman's correlation coefficients, Mann-Whitney U test, and Chi-square test were used to analyze data.

Results

The study cohort consisted of 70 men (35%) and 130 women (65%). The average age was 51.65 ± 14.64 years (52 years). The mean neutrophil count was 4.6 ± 1.5 (4.29) in the control group and 4.7 ± 2.03 (4.12) in the IBS group. The mean lymphocyte count was 2.3 ± 0.86 (2.21) in the control group and 2.3 ± 0.82 (2.23) in the IBS group, indicating no statistically significant difference (p = 0.732). The mean RDW was measured as 13.62 ± 1.07 (13.4) in the control group and 13.68 ± 1.18 (13.55) in the IBS group, again demonstrating no significant difference (p = 0.915). Mean MCV and MPV values showed no substantial variation between the control and IBS groups (p = 0.649 and p = 0.406, respectively).

Conclusion

While this study did not yield statistically robust outcomes, it underscores the potential of utilizing neutrophil-to-lymphocyte ratio (NLR), RDW, and MPV as adjunctive diagnostic markers for IBS. These routine and cost-effective parameters could enhance the diagnostic process, especially in cases with suspected IBS. Continued research is essential to unravel their complete diagnostic potential and clinical applicability.

## Introduction

Irritable bowel syndrome (IBS) represents a prevalent functional gastrointestinal disorder characterized by chronic abdominal pain related to defecation and variable bowel habits, causing considerable diminishment in the individual's quality of life [1]. This syndrome has been referred to by various terms, including terms such as "colonic spasm," "neurogenic IBS colitis," "irritable colon," "unstable colon," "nervous colon," "spastic colon," and "spastic colitis" [2,3].

Despite being largely recognized as a functional disorder, recent investigations have unveiled compelling evidence endorsing the pivotal role of enteric inflammatory processes in the pathogenesis of IBS [4]. Underlying mechanisms encompass mucosal inflammation, immune activation within the mucosa, alterations in intestinal permeability, shifts within the intestinal microbiota, and alterations resulting from post-infectious events [5,6]. The prevalence of IBS is estimated at 11.2% worldwide, exhibiting variations across different geographical regions [7]. Moreover, its prevalence is significantly higher in females, wherein the severity of symptoms significantly impacts their quality of life and daily functioning [7,8]. Incidence rates of IBS incidence rates decline progressively after the age of 50 [7-9].

The substantial burden imposed by IBS on healthcare systems is evident from its propensity to prompt a plethora of hospital visits, superfluous diagnostic evaluations, therapeutic interventions, and even surgical procedures before arriving at a conclusive diagnosis. Furthermore, the efficacy of these interventions is frequently suboptimal, thereby leading to elevated healthcare costs and reduced workforce productivity. Consequently, IBS constitutes a notable healthcare challenge on a global scale [10,11].

The role of platelets in the context of inflammation-related disorders is now well-established within scientific discourse [12,13]. Mean platelet volume (MPV), widely employed as a marker for platelet functionality, not only reflects bone marrow production rates but also platelet activation status [14]. While the study of MPV has been directed toward inflammatory bowel diseases, its exploration in the context of functional gastrointestinal disorders remains a relatively uncharted domain [15,16].

Red cell distribution width (RDW) is indicative of variations in the size of red blood cells. Pertinently, augmented RDW values have been correlated with disorders extending beyond anemia, signifying a broader clinical relevance [17,18]. Analogous to MPV, RDW has been identified as an inflammatory marker among individuals afflicted with inflammatory bowel disease [19,20]. However, its significance in the milieu of functional gastrointestinal disorders remains to be elucidated.

The neutrophil-to-lymphocyte ratio (NLR), which is calculated from the values obtained from complete blood counts, stands as a straightforward and dependable indicator of the inflammatory status within the body. This ratio reflects the balance between neutrophils, which are key players in the body's acute inflammatory response, and lymphocytes, which are essential components of the immune system responsible for regulating and controlling inflammation. The NLR's simplicity and reliance on routine blood tests make it a valuable tool for assessing the degree of inflammation in various medical conditions. Elevated NLR values are generally associated with a more pronounced inflammatory response, and this metric has been recognized as a potential prognostic marker in a range of disorders, highlighting its significance in gauging the body's immune and inflammatory dynamics [14,17,21].

The most recent diagnostic criteria for IBS are encapsulated within the ROME IV criteria, which were introduced in 2016 [22].

ROME IV IBS diagnostic criteria

It includes the symptomatic duration of at least six months preceding the diagnosis, characterized by recurring abdominal pain averaging at least once weekly within the preceding three months, concomitant with two or more of the ensuing features: correlated with defecation (may exhibit amplification or remain unaffected by defecation), concurrent with alterations in stool frequency, and accompanied by modifications in stool form or appearance.

Given the scarcity of investigations concerning hemogram parameters in IBS patients, this retrospective study endeavors to ascertain whether a discriminative diagnosis could be achieved for this intricate malady through the utilization of routine, cost-effective, and readily available blood parameters.

Additionally, the study aims to ascertain whether a discernible relationship exists between hemogram parameters (NLR, RDW, and MPV) and IBS.

## Materials and methods

We conducted a retrospective analysis on patients who attended the outpatient clinic of Hitit University Erol Olçok Teaching and Research Hospital during the period of January 1, 2023, to May 1, 2023. The patients were diagnosed with IBS based on the ROME IV diagnostic criteria. All patients underwent thorough assessments to exclude any presence of psychiatric disorders or organic bowel pathologies. Individuals under the age of 18 and those with established hematological, oncological, or gastrointestinal conditions were excluded from the study. This led to the inclusion of a total of 100 patients with IBS. Concurrently, a cohort of 100 healthy individuals who visited the general surgery outpatient clinic for routine examinations was included as the control group. Importantly, none of the subjects in either the study or control groups had a history of medication intake, such as aspirin, that could potentially impact platelet function.

For all participants, encompassing both patients and controls, comprehensive data were extracted from the hospital's database, encompassing age, gender, serum white blood cell (WBC) count, neutrophil count, lymphocyte count, hemoglobin level, RDW, mean corpuscular volume (MCV), MPV, and platelet count. The study protocol was granted approval by the Ethics Committee on Clinical Research of Hitit University.

Statistical analysis

The entirety of statistical analyses was performed using IBM SPSS for Windows version 26.0 (IBM Corp., Armonk, NY). Descriptive statistics were articulated in terms of counts and percentages for categorical variables, while numerical variables were reported as means ± standard deviations (with corresponding medians). The normality of data distribution was assessed using the Shapiro-Wilk test. Pearson's or Spearman's correlation coefficients were employed, as appropriate, to ascertain the relationships between variables. Mann-Whitney U test was employed to compare numerical variables, including age, serum WBC count, lymphocyte count, hemoglobin level, RDW, MCV, MPV, and platelet count, between the distinct groups. Meanwhile, the comparison of gender distribution between groups was conducted using the Chi-square test. Statistical significance was inferred at a threshold of p < 0.05.

## Results

The study cohort comprised 200 individuals, consisting of 70 men (35%) and 130 women (65%). The mean age of the participants was 51.65 ± 14.64 years (median: 52 years). Within this cohort, the mean WBC count was 7.58 ± 2.01 (median: 7.16), while the mean neutrophil count was 4.65 ± 1.78 (median: 4.35), and the mean lymphocyte count was 2.3 ± 0.84 (median: 2.22). Additionally, the mean hemoglobin value was 14.05 ± 1.73 (median: 14.0), the mean RDW was 13.65 ± 1.12 (median: 13.5), the MCV was 87.46 ± 5.86 (median: 88.35), the MPV was 9.99 ± 1.28 (median: 9.8), and the mean platelet count was 270.98 ± 71.46 (median: 269) among the participants.

Upon comparing the control and IBS groups, it was observed that 66% of patients in the control group were women, and this proportion was 64% in the IBS group, signifying no statistically significant difference (p = 0.767). The mean age was 51.18 ± 14.37 (median: 51) in the control group and 52.12 ± 14.96 (median: 53) in the IBS group, displaying no substantial difference (p = 0.481).

Similarly, the mean WBC count was 7.54 ± 1.79 (median: 7.2) in the control group and 7.62 ± 2.21 (median: 6.9) in the IBS group, demonstrating no significant variation (p = 0.582). Likewise, the mean neutrophil count was 4.6 ± 1.5 (median: 4.29) in the control group and 4.7 ± 2.03 (median: 4.12) in the IBS group, revealing no notable distinction (p = 0.64). The mean lymphocyte count was 2.3 ± 0.86 (median: 2.21) in the control group and 2.3 ± 0.82 (median: 2.23) in the IBS group, indicating no statistically significant divergence (p = 0.732). The mean hemoglobin value was 14.07 (median: 14) in the control group and 14.02 ± 1.85 (median: 14.01) in the IBS group, further highlighting no significant discrepancy (p = 0.901).

Moreover, the mean RDW was identified as 13.62 ± 1.07 (median: 13.4) in the control group and 13.68 ± 1.18 (median: 13.55) in the IBS group, underscoring a lack of substantial difference (p = 0.915). Similarly, no statistically significant distinction was detected in the mean MCV and MPV values between the control and IBS groups (p = 0.649 and p = 0.406, respectively). The mean platelet count was 270.03 ± 70.21 (median: 269.5) in the control group and 271.93 ± 73.03 (median: 268.5) in the IBS group, confirming the absence of significant variation (p = 0.846) (Table 1).

**Table 1:** All patients' data and comparison between groups WBC: White blood cell; RDW: Red blood cell distribution width; MCV: Mean corpuscular volume; MPV: Mean platelet volume.

Variables		All Participants (n = 200)	Control Group (n = 100)	IBS Group (n = 100)	Statistical Significance
Gender	Male	70 (35%)	34 (34%)	36 (36%)	0.767
	Female	130 (65%)	66 (66%)	64 (64%)	
Age		51.65 ± 14.64 (52)	51.18 ± 14.37 (51)	52.12 ± 14.96 (53)	0.481
WBC		7.58 ± 2.01 (7.16)	7.54 ± 1.79 (7.2)	7.62 ± 2.21 (6.9)	0.582
Neutrophil		4.65 ± 1.78 (4.25)	4.6 ± 1.5 (4.29)	4.7 ± 2.03 (4.12)	0.640
Lymphocyte		2.3 ± 0.84 (2.22)	2.3 ± 0.86 (2.21)	2.3 ± 0.82 (2.23)	0.732
Hemoglobin		14.05 ± 1.73 (14)	14.07 ± 1.62 (14)	14.02 ± 1.85 (14.01)	0.901
RDW		13.65 ± 1.12 (13.5)	13.62 ± 1.07 (13.4)	13.68 ± 1.18 (13.55)	0.915
MCV		87.46 ± 5.86 (88.35)	87.5 ± 5.31 (88.15)	87.42 ± 6.39 (88.6)	0.649
MPV		9.99 ± 1.28 (9.8)	10.12 ± 1.36 (10.05)	9.86 ± 1.19 (9.75)	0.406
Platelets		270.98 ± 71.46 (269)	270.03 ± 70.21 (269.5)	271.93 ± 73.03 (268.5)	0.846

## Discussion

IBS represents a heterogeneous disorder characterized by diverse clinical manifestations and a complex underlying pathophysiology. Epidemiological investigations have highlighted potential factors such as inflammation, dietary components, genetics, and environmental influences in the development of this condition [23]. Despite efforts to diagnose IBS, established laboratory parameters remain elusive. Routine laboratory assessments often yield unremarkable results in IBS patients, although previous studies suggest the presence of inflammatory changes in colonic mucosa and alterations in motility [24,25]. Additionally, increased enterochromaffin (EC) cells, interleukin-1β expression, and T lymphocytes have been identified in the lamina propria of colonic mucosa in individuals with IBS [26].

Laboratory parameters have gained importance within scoring systems for diagnostic purposes. Contrary to the past belief of the absence of morphological or biochemical abnormalities in IBS, recent studies have revealed the role of inflammatory alterations in its pathophysiology [27]. Various investigations have explored potential diagnostic parameters for IBS, covering a wide range of aspects [19]. Despite studies on individual parameters within complete blood counts, there is a scarcity of retrospective studies examining the entire spectrum of these parameters, highlighting the significance of our comprehensive approach. However, our study did not identify statistically significant differences in the NLR, MPV, and RDW levels between IBS patients and control subjects.

The exploration of novel inflammatory biomarkers has gained momentum. Neutrophils, lymphocytes, RDW, MPV, and platelets play crucial roles in inflammatory processes [20,28]. Literature reports have established associations between NLR and states of inflammation. NLR, calculated from complete blood counts, is a widely recognized inflammatory marker, with elevated values predicting adverse clinical outcomes [29]. Its predictive utility extends to various clinical conditions. Moreover, elevated NLR has been linked to the severity of mucosal disease in ulcerative colitis [30].

MPV offers insight into the size of circulating platelets. Notably, augmented MPV values often herald platelet activation, a recurrent feature of inflammatory states [31]. In line with this, Aktaş et al. reported elevated MPV values among IBS patients [19]. Contemporary scientific discourse has witnessed a surge in studies scrutinizing the implications of both RDW and MPV on mortality outcomes [32]. Although traditionally associated with anemia assessment, mounting evidence underscores heightened mortality risks in the context of elevated RDW levels across diverse conditions encompassing sepsis, trauma, and cardiovascular maladies [33]. Similarly, Song et al. established a correlation between RDW and disease severity within inflammatory bowel diseases, noting escalated RDW values in ulcerative colitis and Crohn's disease [20]. This aligns with the findings by Arhan et al., who demonstrated heightened RDW and MPV levels among inflammatory bowel disease cohorts relative to healthy subjects [34]. Additionally, Çakal et al. illuminated reductions in hemoglobin and MCV levels concomitant with a surge in RDW levels with escalating disease severity in both ulcerative colitis and Crohn's disease, advocating the role of these indices in early diagnosis [35]. Correspondingly, Zubcevic et al. delineated augmented platelet counts but unaltered MPV levels with the progression of Crohn's disease, corroborating the active involvement of platelets in inflammatory processes [36].

A prior study documented heightened RDW levels in 8.3%, 63.3%, and 45.7% of cases of IBS, Crohn's disease, and ulcerative colitis, respectively, positioning RDW as a prospective gauge of disease severity in both Crohn's disease and ulcerative colitis.

Regrettably, our study bears certain limitations. Primarily, its retrospective design poses an inherent constraint. Furthermore, the limited sample size necessitates caution in generalizing findings to the entirety of IBS patients. It is imperative to acknowledge the diagnostic intricacies and challenges that pervade IBS, which is further complicated by its variable follow-up trajectories and treatment response assessments.

Our inquiry constitutes a meticulous exploration of readily accessible blood parameters, obviating the need for supplementary tests. Moreover, it is noteworthy for its robust IBS diagnosis, encompassing patient populations exhibiting analogous symptoms. The strength of our study is further accentuated by the rarity of comprehensive investigations evaluating the aforementioned parameters collectively within the IBS context.

## Conclusions

We posit that the incorporation of NLR, RDW, and MPV may furnish valuable assistance in the diagnostic realm of IBS. Although the diagnosis of IBS is conventionally anchored in historical and clinical considerations, these unassuming and cost-effective hematological parameters hold potential to complement the diagnostic process, particularly in cases where suspicion prevails.

Admittedly, while our study did not culminate in statistically robust outcomes, its findings will undoubtedly serve as an illuminating beacon for analogous investigations, beckoning the exploration of elementary and economically accessible parameters that hold promise in expediting the diagnostic pursuit of IBS within clinical practice.
